# Supplementary material for: Functionalized GO/Hydroxy-Terminated Polybutadiene Composites with High Anti-Migration and Ablation Resistance Performance
Source: Polymers (Basel). 2022 Aug 15;14(16):3315. doi: 10.3390/polym14163315 (PMC9414271; doi:10.3390/polym14163315)
Supplement: Supplementary file 1 [file polymers-14-03315-s001.zip › polymers-1870398-supplementary.pdf]

polymers

Supporting Information

Functionalized GO/Hydroxy-Terminated Polybutadiene Composites with High Anti-Migration and Ablation Resistance Performance

Shuai Yin [1], Zhehong Lu [1], Haoran Bai [2], Xinyang Liu [3], Hao Li [3] and Yubing Hu [1,*]

[1] National Special Superfine Powder Engineering Research Center of China, Nanjing University of Science and Technology, Nanjing 210014, China
[2] College of Materials Science and Engineering, Shenyang University of Technology, Shenyang 110870, China
[3] College of Materials Science and Engineering, Nanjing Tech University, Nanjing 211816, China
* Correspondence: hyb@njust.edu.cn

Experimental

Materials

Hydroxyl-terminated polybutadiene (HTPB, 99.9%, hydroxyl value ≥ 0.1mol/100g) was purchased from Tianjin Siense Biochemical Technology Co., Ltd., China. Isophorone diisocyanate (IPDI, ≥98.0%)were purchased from Shanghai Aladdin Company, China. Dioctyl Sebacate (DOS, ≥97.0%) was purchased from Shanghai Macleans Biochemical Technology Co., Ltd., China. Graphene oxide (GO) was prepared by a modified Hummers method.

Preparation of IPDI functionalized GO

First, 6 g graphite powder and 3 g $NaNO_3$ were added to a beaker containing 150 mL H_2SO_4 in an ice bath. Then, 18 g $KMnO_4$ was added in batches. The temperature of the water bath was raised to 35 °C. After 2 h, 200 mL of deionized water was added, and stirring was continued for another 2 h. The temperature was then raised to 98 °C, and the solution was diluted with 400 mL of deionized water. 30% H_2O_2 was added dropwise until the solution turned bright yellow. Metal ions were washed away by dilute HCl. The solution was centrifuged and washed until neutral and then lyophilized for later use.

The lyophilized GO (0.2 g) was weighed, and a 1 mg/mL suspension was prepared using anhydrous DMF. The suspension was dispersed ultrasonically for 3 h and stirred at 80 °C. Then, 1 g of IPDI was added dropwise to the reaction solution, and then a drop of DBTL was added. The reaction proceeded for 5 h under N_2 protection. After the reaction, the product was filtered, and unreacted IPDI was washed away with DMF. The product was dried for later use.

Migration kinetics

As an important migration kinetic parameter, the migration coefficient can vividly describe the speed of the migration process, and the migration data is further processed to obtain the migration coefficient. According to Fick's second law [2]:

$$\frac{\partial c}{\partial t} = D\frac{\partial^2 c}{\partial x^2}$$

Assuming that the migration is unidirectional under non-steady state conditions, with a constant diffusion coefficient, the amount of migration through the sample:

$$\frac{M_t}{M_\infty} = 1 - \frac{8}{\pi^2}\sum_{n=0}^{+\infty}\frac{1}{(2n+1)^2} exp\left[-\frac{D(2n+1)^2\pi^2}{{d_i}^2}t\right]$$

Simplified:

$$\frac{M_t}{M_\infty} = \frac{4}{d_i}\left(\frac{Dt}{\pi}\right)^{1/2}$$

Where M_t is the amount of migration at time t, and M_∞ is the amount of migration at equilibrium.

List the expression formula of the required data and convert it into the existing parameter form:

$$m_0 = \rho_i d_i A,\quad M_\infty = \frac{1}{2}\rho d_i A t(x),\quad M_t = \Delta m = \frac{4}{d} t^{1/2}\left(\frac{D}{\pi}\right)^{1/2} \cdot M_\infty$$

$$\frac{\frac{\Delta m}{m_0}}{\left(t^{\frac{1}{2}}\right)} = \frac{2\rho t(x)}{\rho_i d_i}\left(\frac{D}{\pi}\right)^{\frac{1}{2}}$$

Derivative of the differential to obtain the calculation formula of the migration coefficient [3]:

$$\frac{d}{d\sqrt{t}}\left(\frac{\Delta m}{m_0}\right) = \frac{2\rho}{\rho_i d_i}\left(\frac{D}{\pi}\right)^{1/2}$$

Where Δm is the amount of DOS migrated into the liner at time t, m0 is the initial mass of the liner, ϱ is the density of DOS 0.918g/cm^3, ϱi is the liner density, and di is the thickness of the liner. Use $t_{1/2}$ and $\Delta m/m_0$ as the abscissa and ordinate respectively to draw the point diagram, and the linear slope obtained after linear fitting can be calculated by the formula of Fick's second law to calculate the migration coefficient.
